# Transmission-Blocking Strategies Against Malaria Parasites During Their Mosquito Stages

**DOI:** 10.3389/fcimb.2022.820650

**Published:** 2022-02-16

**Authors:** Shasha Yu, Jing Wang, Xue Luo, Hong Zheng, Luhan Wang, Xuesen Yang, Ying Wang

**Affiliations:** ^1^Department of Tropical Medicine, College of Military Preventive Medicine, Army Medical University, Chongqing, China; ^2^Department of Thoracic Surgery, Xinqiao Hospital, Army Medical University, Chongqing, China

**Keywords:** malaria, transmission-blocking, drug, transmission-blocking vaccine, *Wolbachia*, mosquito intestinal flora

## Abstract

Malaria is still the most widespread parasitic disease and causes the most infections globally. Owing to improvements in sanitary conditions and various intervention measures, including the use of antimalarial drugs, the malaria epidemic in many regions of the world has improved significantly in the past 10 years. However, people living in certain underdeveloped areas are still under threat. Even in some well-controlled areas, the decline in malaria infection rates has stagnated or the rates have rebounded because of the emergence and spread of drug-resistant malaria parasites. Thus, new malaria control methods must be developed. As the spread of the *Plasmodium* parasite is dependent on the part of its life cycle that occurs in mosquitoes, to eliminate the possibility of malaria infections, transmission-blocking strategies against the mosquito stage should be the first choice. In fact, after the gametocyte enters the mosquito body, it undergoes a series of transformation processes over a short period, thus providing numerous potential blocking targets. Many research groups have carried out studies based on targeting the blocking of transmission during the mosquito phase and have achieved excellent results. Meanwhile, the direct killing of mosquitoes could also significantly reduce the probability of malaria infections. Microorganisms that display complex interactions with *Plasmodium*, such as *Wolbachia* and gut flora, have shown observable transmission-blocking potential. These could be used as a biological control strategy and play an important part in blocking the transmission of malaria.

## 1 Introduction

Malaria is still the most prevalent parasitic disease affecting humans globally, with approximately 228 million cases and 405,000 deaths per year (Ashour and Othman, 2020). Although it has been controlled well in many regions, in some underdeveloped areas, especially in southern Africa, more than one billion people are at risk. The current successes mainly rely on treatment with artemisinin-based combination therapy (ACT), the indoor residual spraying of insecticides, and insecticide-treated mosquito nets. However, existing malaria control measures have become less effective owing to the emergence of multidrug-resistant parasites and insecticide-resistant mosquitoes, resulting in a recent pause and even a reverse in the reduction of malaria infections (Bhatt et al., 2015; Ippolito et al., 2018; Romoli and Gendrin, 2018; Shaw and Catteruccia, 2019; Moyes et al., 2020). As a warning, it was reported that resistance to frontline artemisinin-based drugs was spreading in the Greater Mekong Subregion of Southeast Asia. Recent studies have shown that similar artemisinin-resistant strains have appeared in Africa, which may be a dangerous signal for global malaria control (Lubell et al., 2014; Slater et al., 2016; Stokes et al., 2021; L’Episcopia et al., 2021; Owoloye et al., 2021). Therefore, to prevent the resurgence of malaria in well-controlled regions and ensure the progressive decline of malaria infection in high-incidence areas, it is necessary to find more effective and safer malaria control strategies.

Thus far, the development of new drugs is still an important method of malaria control, but given the current situation of increasing drug resistance, this method may face huge pitfalls and result in relatively small benefits. Many countries and regions are committed to eradicating malaria, rather than simply controlling its incidence; thus, safe and effective malaria vaccines could be a crucial tool. At present, the development of malaria vaccines mainly consists of three types, targeting different stages of the malaria parasite life cycle: (1) pre-erythrocytic vaccines targeting the sporozoites and liver stages; (2) blood-stage malaria vaccines targeting the asexual blood stages; and (3) transmission-blocking vaccines (TBVs) targeting the sexual stages and mosquito midgut antigens (Carter et al., 2000; Richie and Saul, 2002; Goodman and Draper, 2013). Currently, the pre-erythrocytic vaccines whose main candidate antigen is circumsporozoite protein act to prevent the appearance of the hepatic and erythrocytic stages *via* attacking the sporozoites (Molina-Franky et al., 2020). In addition, the main liver-phase vaccine RTS, S and blood-phase vaccine, whose candidate protein is the merozoite antigen, are mainly designed to prevent the appearance of the erythrocytic stage to provide benefits for clinical malaria (Abdulla et al., 2008; Bejon et al., 2008). Therefore, to block the spread of malaria and eliminate the possibility of malaria infection, TBVs, which mainly target proteins related to the developmental stage after the gametocyte stage (processes that occur in mosquitoes), may be an excellent choice (Wang et al., 2017). In addition, it was predicted that TBV administration could reduce child mortality even in areas of high endemicity (Smith et al., 2001). TBVs can also slow the spread of mutant parasites, prolonging the efficacy of antimalarial drugs and vaccines (Kaslow, 2002). The study of a multi-stage combined vaccine facilitated more extensive application prospects for TBVs (Draper et al., 2018; Yusuf et al., 2019).

However, it is inevitable that the development of vaccines is an extremely long process that requires time to overcome many difficulties and technical obstacles. In fact, several studies have shown that some biological control strategies could also provide solutions to blocking the transmission of malaria (Caragata et al., 2020). Throughout the life cycle of malaria, in addition to the interactions between the malaria parasites and their hosts, many microorganisms also play integral and unique roles in the parasitic life cycle. An increasing number of research groups are exploring how to use the interaction between malaria parasites and various microorganisms to develop malaria control strategies during the growth period (Dahmana and Mediannikov, 2020; Gabrieli et al., 2021). Notably, the technical obstacles of this transmission-blocking strategy may be less difficult to overcome than those of vaccine development, but it is essential to pay great attention to its ecological impact.

In this review, we mainly focus on transmission-blocking drugs, the results arising from the design of TBVs and the challenges that need to be overcome in the subsequent period, as well as summarizing existing biological blocking strategies, which are mainly based around *Wolbachia* and the mosquito intestinal flora. We believe that these findings may provide a reference for the development of malaria transmission-blocking strategies in the future.

## 2 Transmission-Blocking Drugs for *Plasmodium*

Although ACT treatment is very effective in killing the asexual blood stage malaria parasites and curing patients, it cannot completely remove mature gametocytes from the blood. Therefore, malaria patients treated with ACT remain infectious to blood-sucking mosquitoes for 1–3 weeks (WWARN Gametocyte Study Group, 2016). Primaquine is currently the only malaria transmission-blocking drug recommended by the WHO. It can effectively remove mature gametocytes from the blood, but it is not being widely used owing to its safety issues in glucose-6-phosphate dehydrogenase-deficient patients (White et al., 2012). Therefore, in recent years, many studies have been exploring new drugs to solve this problem. Some progress has been made in the improvement of the structure of primaquine itself and in the identification of new possible blocking drugs from natural products derived from plants and microorganisms (Moyo et al., 2020; Boechat et al., 2020).

In addition to killing gametocytes, the spread of malaria could also be blocked by targeting other parasitic stages that occur in mosquitoes (the gametes, zygotes, oocytes, and oocysts) (Smith et al., 2014). Atovaquone and a combination of atovaquone and proguanil were reported to be capable of reducing mosquito infectivity, hence blocking malaria transmission, *via* inhibiting ookinete formation and oocyst maturation (Butcher and Sinden, 2003; Vos et al., 2015; Azevedo et al., 2017). Many other drugs have also been found to have similar properties (Wadi et al., 2019). However, a major problem in blocking the spread of malaria in this way is that the delivery of the drugs is indirect. This means that the drugs must remain in the patient’s blood for an extended period at an effective concentration, thus greatly inhibiting the development of this transmission-blocking strategy (Wadi et al., 2018). Moreover, the sporogonic stages (gametes, zygotes, ookinetes, oocysts and sporozoites) themselves are still insufficiently studied as drug targets for now. However, antigens from these stages are being extensively investigated as targets for TBVs. (Sauerwein and Bousema, 2015; Birkett, 2016).

## 3 Transmission-Blocking for *Plasmodium* Sexual Stages

**Figure 1:**
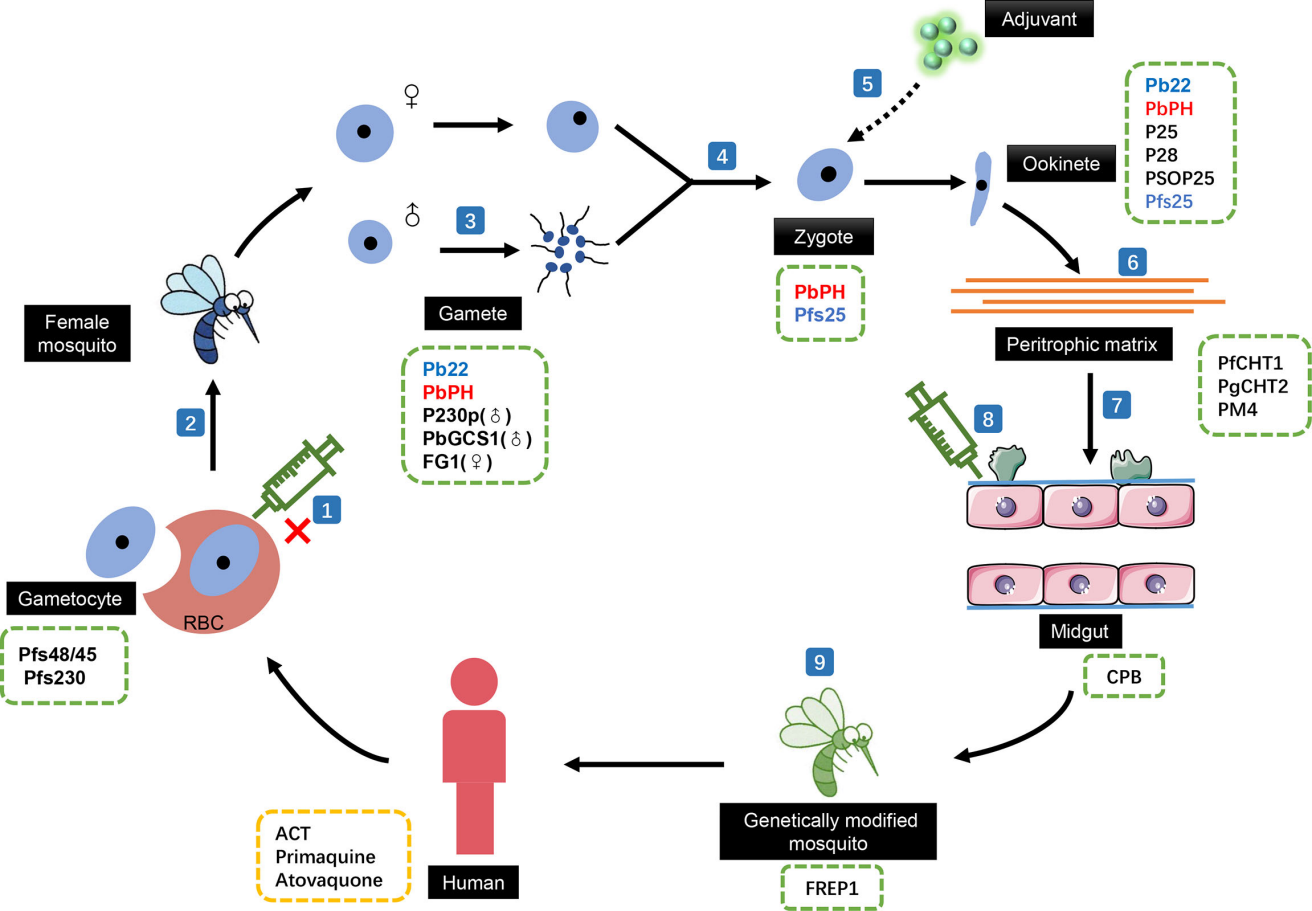
The effect of transmission-blocking vaccines (TBVs) in the sexual reproductive stages of malaria. (1) TBV cannot act on gametocytes that have not escaped from red blood cells. The development of TBVs based on the (2) surface proteins of the gametocytes, (3) male gamete-related targets, and (4) fertilization process-related proteins can effectively block the development process of malaria in mosquito. (5) The lack of inherent antigenicity means that TBVs based on ookinete surface proteins need adjuvants in actual use. Blocking the ability of motility ookinetes to break through the (6) peritrophic matrix and the (7) physical and immune barriers of the midgut to prevent its colonization in the midgut is another development direction of TBVs. (8) TBVs targeting mosquito midgut proteins can also effectively block ookinete colonization. (9) Directly genetically modified mosquitoes to prevent the development of ookinetes. Candidate target proteins for transmission blocking antibodies in different stages of malaria life cycle were listed in green dotted boxes. Anti-malaria drugs that could influence the transmission were listed in yellow dotted box. ACT, artemisinin-based combination therapy; Pfs48/45, *Plasmodium falciparum* gametocyte surface protein; Pfs230, *P. falciparum* gametocyte surface antigen; Pb22, a conserved protein (PBANKA_0305900) in *Plasmodium berghei*; PbPH, *P. berghei* pleckstrin homology gene; PbGCS1, *P. berghei* Generative Cell Specific 1; FG1,female gamete peptide 1; Pfs25, *P. falciparum* sexual-stage surface protein; P25, the major surface proteins of *Plasmodium* ookinetes; P28, Ookinete surface antigen-like protein; PSOP25, putative secreted ookinete protein 25; PfCHT1, *P. falciparum* chitinase; PgCHT2, *P. gallinaceum* chitinase; PM4, plasmepsin 4; CPB, Carboxypeptidase B; FREP1, Fibrinogen-related protein 1.

Malaria causes alarming morbidity and mortality in more than 100 countries worldwide. On studying the vaccine development of malaria, it was observed that pre-erythrocytic vaccines can only protect residents in areas of low-endemicity from becoming infected, and blood-stage malaria vaccines have been designed to reduce the severity of the clinical disease (Wang et al., 2017). Thus, TBVs, which aim at stopping the spread of malaria and eliminating the possibility of infection (**Figure 1**), have received increasing attention (Kaslow, 2002).

### 3.1 Transmission-Blocking From Infected Patients to Mosquitoes

Throughout malaria infection progression, the asexual forms are responsible for clinical malaria, while the sexual stages are responsible for continued transmission *via* mosquitoes. In the erythrocytic stage, < 1% of *Plasmodium* parasites commit to forming gametocytes in infected red blood cells. After male and female gametocytes are ingested in a mosquito blood meal, they break out of their red blood cells and initiate the sexual stage of parasite development. Therefore, from a life history perspective, blocking malaria gametocytes from transferring to mosquitoes is the first important step in reducing global malaria transmission.

So far, most of the gametocyte surface proteins considered as TBV candidates have been from the six-cysteine motif (6-CM) protein family, and the most studied members of this family are Pfs48/45 and Pfs230 (Saul, 2007). Knockout experiments have demonstrated that these two proteins are essential for fertilization after the gametocyte enters the mosquito (van Dijk et al., 2001; Eksi et al., 2006). Although antibodies targeting Pfs230 could prevent oocyst formation in mosquito through standard membrane-feeding tests and/or direct membrane-feeding tests, the applied research seems to have stagnated because the functional antibodies used for the investigation can only be produced *in vivo* now (Farrance et al., 2011; Miura et al., 2013; Kapulu et al., 2015). Producing recombinant Pfs230 proteins *in vitro* remains challenging. This is mainly because Pfs230 is a >300 kDa protein consisting of 14 CM domains and both the size and cysteine-rich nature of the molecule have hampered its production as an intact protein. Therefore, researchers have been attempting to determine the domain that is responsible for the blocking ability of this protein for future vaccine development. A study produced multiple fragments of the Pfs230 molecule using a eukaryotic wheat germ cell-free expression system. In subsequent experiments, they found that protein fragments containing CM domain 1 displayed strong transmission-blocking effects, while antibodies generated using constructs without CM domain 1 showed no inhibition. This suggests that CM domain 1 may be an excellent choice (Tachibana et al., 2019). In addition, studies have reported that liposome vaccine adjuvant admixed with Pfs230 fragments could trigger a stronger blocking effect (Huang et al., 2020). Similarly, although the molecular size of Pfs48/45 is appropriate, the correct and efficient production of recombinant vaccines is still an urgent problem that needs to be solved. Multiple studies tackling this issue have also achieved good results, either using DNA plasmid encoding after codon optimization or endo H enzymatic deglycosylation in plants. Both significantly enhanced the immunogenicity of recombinant Pfs48/45 and strengthened its transmission-blocking ability (Datta et al., 2017; Mamedov et al., 2019; Lee et al., 2020). In addition, a study reported that a Pfs230–Pfs48/45 chimeric malaria transmission-blocking vaccine could induce a stronger blocking effect than a single protein and identified a modified construct (ProC6C) as a possible solution to the low yield of this vaccine (Singh et al., 2020; Singh et al., 2021).

In addition, this vaccine is unusual in that the antibodies cannot kill the gametocytes in the red blood cells of malaria patients; it is only during the mosquito’s blood meal from an infected host that the antibodies can efficiently act on the gametes after they have emerged from their host red blood cells, thus preventing infection in the mosquito and halting the spread of malaria (Carter and Chen, 1976; Gwadz, 1976; Saul, 2007). In the Makoni district of Zimbabwe (an area of low to modest malaria transmission), enzyme-linked immunosorbent assay revealed the prevalence (64% positivity at 1:500 dilution in 66 randomly selected plasma samples) of antibodies against recombinant Pfs48/45 (mean A405 nm = 0.53, CI = 0.46–0.60) and Pfs47 (mean A405 nm = 0.91, CI = 0.80–1.02), antigens specific to the sexual stages of the parasite. A mosquito membrane-feeding test demonstrated that samples positive for the Pfs48/45 antibody in the enzyme-linked immunosorbent assay had the ability to reduce malaria transmission (Paul et al., 2016). These results indicate that there may be considerable natural immune antibodies in the infected population that play a role in transmission-blocking, resulting in a low local prevalence. Therefore, identifying the antibodies against gametocyte-expressed proteins produced by natural immunity may accelerate the progress of vaccine development.

Furthermore, intact mature gametocytes do not undergo phagocytosis mediated by monocytes, benefiting malaria transmission. However, red blood cells containing gametocytes that are not transmitted to mosquitoes during infection will die and then be eliminated by phagocytic cells. Therefore, the analysis of the molecular mechanisms in this phagocytic process and subsequent immune response may help to identify more targets for transmission-blocking strategies (Bansal et al., 2016).

### 3.2 Transmission-Blocking for Gametocytes Fertilized Into Zygotes

When gametocytes are ingested by mosquitoes, they transform into gametes as the environment changes (Kehrer et al., 2016). Each female gametocyte forms a single immotile macrogamete, while male gametocytes generate up to eight flagella-like microgametes in a process called exflagellation. Subsequently, the fully differentiated gametes need to dissolve the inner parasitophorous vacuole membrane and the outer erythrocyte membrane to form diploid zygotes, which then continue to develop and mature in mosquitoes (Paul et al., 2002; Sologub et al., 2011; Deligianni et al., 2013). In general, the fertilization process lacks the protection of the host cell membrane and involves many cells and biochemical processes, providing plenty of potential drug targets (Sinden et al., 2012). Therefore, malaria-blocking strategies aimed at the fertilization process have received increasing attention.

A recent mathematical model analysis showed that the human antibodies ingested by mosquitoes were effective at inhibiting fertilization in mosquito midgut, resulting in a decrease in the density of oocysts (Teboh-Ewungkem et al., 2021). This indicates that antibodies targeting gamete proteins may be a potential strategy for transmission-blocking. While female gametocytes only have to leave their red blood cells to become gametes, male gametogenesis also includes three rounds of mitosis and flagellum construction to produce eight gametes (Schall, 2000; Reece et al., 2008). Thus, male gamete-related targets have become the focus of TBV development. A conserved protein of 22 kDa in *Plasmodium berghei*, named Pb22, is located on the plasma membrane of gametes and ookinetes during gamete-to-ookinete development. A recent study showed that the exflagellation of male gametes (~89%) and ookinete numbers (~97%) were significantly reduced after Pb22 was knocked out, and these defects were rescued in parasites when Pb22 was restored. Further analysis showed that the defects of the Pb22 knockout (KO)line were limited to the male gametes, and the female gametes in Pb22-KO line were fertile at the wild-type level, indicating that Pb22 was indeed a key protein in the development of male gametes. Of the Pb22-KO line male gametocytes, 30% failed to assemble axonemes, whereas ~ 48.9% formed flagella but failed to egress from the host erythrocytes (Liu et al., 2021). In addition, antibodies against the highly conserved *Plasmodium berghei* pleckstrin homology gene PbPH that is localized on the surface of gametes, zygotes, and ookinetes significantly inhibited *the* exflagellation of male gametocytes and the formation of ookinetes in a concentration-dependent manner (Kou et al., 2016). P230p, which is expressed only in male gametocytes and gametes, is another potential target. Studies have shown that, after knocking out P230p, male gametes can normally form outer flagella but cannot attach to the red blood cell membrane, resulting in a significant reduction in zygotes and oocysts (Marin-Mogollon et al., 2018). Moreover, it was previously observed that the knockout of Generative Cell Specific 1 (GCS1) in angiosperms could cause male sterility, while the knockout of PbGCS1 in *Plasmodium* also showed a male sterility phenotype (Hirai et al., 2008). Subsequent studies further demonstrated that GCS1 (also known as Hap2) was indeed a TBV target candidate; TBVs based on GCS1 could trigger a strong transmission-blocking effect (Blagborough and Sinden, 2009; Angrisano et al., 2017; Qiu et al., 2020; Feng et al., 2021). This suggests that research across different species may have inherent connections, which is worthy of in-depth exploration.

Importantly, although male gametes have occupied an important position in the development of TBVs, remarkably little is known about the ecology and behavior of male gametes. Male gametes need to locate and fertilize females in the challenging environment of the mosquito blood meal, and studies have shown that tryptophan metabolites can affect the fertilization process. Meanwhile, a proteomic analysis revealed that glycolysis may be the exclusive energy source for the flagellar beat of male gametes (Talman et al., 2014). Moreover, male gametes may not move randomly, and somehow the female gametes attract the male gametes (Carter et al., 2016). At present, studies on this aspect are rare, but further analysis of the factors affecting the fertilization process, would greatly broaden our understanding and facilitate the development of TBVs.

In fact, aside from male gametes, there are other potential vaccine targets. Although a variety of TBV candidates have been discovered from the study of male gametes, the proteins that interact with these targets are still poorly understood. It has recently been found that the combination of female gamete peptide 1, a peptide that binds specifically to the surface of female gametes, and female gametes interferes with the fertilization of male gametes, thereby significantly reducing the number of oocysts (Vega-Rodriguez et al., 2015).

However, the strategy of blocking the spread of malaria during the fertilization process still has an aspect that cannot be ignored. Studies have shown that *Plasmodium* responds to external transmission pressure by increasing the number of male and female gametocytes. When the density of gametocytes is low, *Plasmodium* can increase the rate of transmission success by increasing the proportion of male gametes (Mitri et al., 2009; Ramiro et al., 2011). This fact is of concern as the TBVs targeting the fertilization process almost all target the male gametes, and the *Plasmodium* may offset the effect of these TBVs by increasing the number of male gametes. Therefore, sex-specific vaccines targeting gametes cannot provide satisfactory blocking effects. An understanding of how the malaria parasite senses external pressure is needed and the mechanism of changing gamete distribution is a key issue that needs to be resolved.

## 4 Transmission-Blocking for the Post-Midgut Developmental Stage

When female macrogametes are fertilized and transformed into motile ookinetes, they need to invade the midgut epithelium of the mosquito. Upon reaching the basal lamina, the motile ookinetes continue to develop into oocysts. Thousands of sporozoites then form in the mature oocysts, enter the hemocoel and invade the salivary glands of the mosquito in preparation for inoculation into new human hosts (Sinden, 1999). This final process of the reproductive development stage of *Plasmodium* is thought to be another critical step. Transmission-blocking strategies targeting this stage have also received significant attention.

The most common transmission-blocking strategy is inhibiting the ability of the motile ookinetes to directly invade the midgut epithelium. In fact, this invasion process is the most difficult step for development of malaria parasites in mosquito because the motile ookinetes need to break through many obstacles for successful colonization. The peritrophic matrix, composed of proteins, glycoproteins, proteoglycans, and chitin, is the first physical barrier faced by the ookinete (Sieber et al., 1991; Moskalyk et al., 1996; Shen and Jacobs-Lorena, 1998). Antibodies targeting chitinase (PfCHT1 or PgCHT2) and a secreting plasma protease (aspartic protease plasmepsin 4, PM4) in *Plasmodium* have been shown to inhibit the passage of ookinetes through the peritrophic matrix, thereby significantly reducing the number of oocysts and the infectivity of malaria (Li et al., 2005; Li et al., 2010). After the peritrophic matrix, motile ookinetes also need to overcome the physical barrier and innate immune defense system of the midgut epithelium (Meister et al., 2005). The highly conserved ookinete surface proteins (P25 and P28) and the putative secreted ookinete protein 25 have been shown to assist the motility of ookinetes that attach and invade the midgut epithelium. Studies have further shown that antibodies targeting these proteins can affect ookinete maturation and oocyst formation in a concentration-dependent manner (Baton and Ranford-Cartwright, 2005; Zheng et al., 2017). However, it should be noted that the lack of inherent antigenicity of Pfs25 leads to the need for a strong human-use-compatible adjuvant for this vaccine in practical application. In a phase I clinical trial, erythema nodosum associated with a Montanide ISA 51 oil-in-water adjuvant was tested in two cases, and the subjects showed frequent local reactogenicity throughout the trial (Wu et al., 2008). Therefore, the development of safer and more efficient adjuvants is urgently needed for this vaccine. A previous study explored the blocking efficiency of alga-produced Pfs25 in combination with four different human-compatible adjuvants (alum, Toll-like receptor 4 agonist glucopyranosal lipid A plus alum, squalene-oil-in-water emulsion, and glucopyranosal lipid A plus squalene-oil-in-water emulsion) and demonstrated that Toll-like receptor 4 agonist in a squalene-oil-in-water emulsion may be a promising adjuvant (Patra et al., 2015). Moreover, the TatD-like DNase of *Plasmodium*, which is considered a conservative protein that plays an important role in immune escape in the asexual stage, is not only expressed during the red blood cell stage, but also throughout the developmental stages of mosquito vectors (Marin-Esteban et al., 2012). Interestingly, the combined immunization of recombinant TatD-like DNase and Montanide ISA51 was found to induce a strong humoral response and weaken the ability of the malaria parasite to break through the midgut innate immune barrier, which significantly prevented the development and transformation of parasites in the midgut of mosquitoes in a mouse model (Wang et al., 2018).

However, vaccines targeting the protein in the reproductive period of *Plasmodium* still have a disadvantage that cannot be ignored. With the use of these vaccines, the malaria parasites are under great selective pressure; thus, they are likely to produce unexpected mutations and escape immune responses (Coutinho-Abreu and Ramalho-Ortigao, 2010). As the developmental stage is also completed under the influence of the mosquito’s system, designs based on mosquito target proteins are a potential method of solving this problem, and some excellent research results have been reported. The design of vaccines based on midgut surface-related proteins is the main exploration direction. Carboxypeptidase B in the midgut of mosquitoes has been demonstrated to be essential for the sexual development of *Plasmodium* in mosquitoes, and several drugs from the Food and Drug Administration, such as NSC-1014, NSC 332670, and aminopterin, have been confirmed to significantly reduce carboxypeptidase B activity (Mongkol et al., 2015). Further improvements to these three drugs may help in the development of TBVs. In addition, antibodies against some conserved proteins in the midgut microvilli also disrupt the development of *Plasmodium* oocysts (Dinglasan et al., 2007; Lecona-Valera et al., 2016).

It is even more surprising that the direct genetic modification of mosquito vectors may be a more promising solution. A recent study has attempted this approach. Fibrinogen-related protein 1 (FREP1), a member of the fibrinogen-related protein family (also known as fibrinogen domain immunolectin), has been shown to be involved in the infection process of *Plasmodium* in mosquitoes (Dong et al., 2006; Dong and Dimopoulos, 2009; Simoes et al., 2017). Previous studies have shown that FREP1 mainly plays a role in the midgut development stage of *Plasmodium* in mosquitoes (Zhang et al., 2015). In this study, researchers used the CRISPR/Cas9 model to simulate mosquitoes lacking FREP1, and the number of oocysts was indeed significantly suppressed in this phenotype when further infected with *Plasmodium*. However, although the exclusion of FREP1 had an excellent blocking effect on the spread of malaria parasites, many issues still need to be resolved. In subsequent observations, the inactivation of FREP1 had a significant detrimental effect on the health of the mosquitoes, including a significantly lower propensity for blood-sucking, lower fecundity and egg hatching rates, retarded pupation times, and reduced longevity after a blood meal (Dong et al., 2018). This suggests that a target gene with lower detrimental health effects may be needed in the future to ensure the survival of the modified mosquitoes.

In particular, the exploration into blocking strategies during this stage need to consider a particular problem. A recent study showed that when the intensity of mosquito infection was extremely high, starvation conditions reduced the activity of RNA polymerase III in the oocysts, leading to a decrease in the growth and maturation rate of the oocysts. However, when the mosquitoes were given another blood meal, the number of oocysts was completely restored (Habtewold et al., 2021). As the mosquitoes were in a highly infected state during the experiment to evaluate the transmission-blocking effect, these results hint that the change of oocysts number after another feeding might be considered another key assessment factor for the realistic transmission-blocking ability of TBVs.

## 5 Transmission-Blocking for Multi-Stage Combination Vaccine

To date, the development of malaria vaccines has been mainly based on a single antigen from different life stages of the malaria parasite. This has raised concerns that single-stage vaccines may be ineffective owing to sequence variability among different parasite isolates, host genetic restrictions of immune responses to specific epitopes, and the short-lived protective immunity induced by some single-antigen vaccines (Shi et al., 2000). Therefore, a multi-stage target combined vaccine is considered a powerful solution. A dual-target vaccine for the asexual and sexual stages has been shown to be feasible; when a fusion protein comprising the *Plasmodium vivax* circumsporozoite and P25 proteins was included in a combination vaccine, significant protective effects (43%) and blocking effects (82%) were observed simultaneously (Mizutani et al., 2014). In addition, several benzimidazole derivative compounds and a series of newly synthesized internal peroxy compounds also exhibited dual effects in the asexual and sexual stages of *Plasmodium* (Miranda et al., 2014; Leshabane et al., 2021). However, combination immunization vaccines targeting different developmental stages in the mosquito phase did not seem to produce synergistic effects. In a previous study, researchers tested the effect of TBVs against both the pre-fertilization antigen Pys48/45 and the post-fertilization antigen Pys25. The results showed that the blocking effect of the composite vaccine was stronger than that of the vaccine based on the Pys48/45 target, but significantly weaker than that of vaccines based on the Pys25 target (Zheng et al., 2016). This suggests that the multi-targeted TBV vaccine may interfere with the induction of antigen-specific antibody responses, and follow-up studies are needed to further explore the reasons for this phenomenon.

## 6 Endosymbiosis in Transmission-Blocking of Malaria

Identifying potential targets based on the life stages of malaria parasites in mosquitoes to design corresponding single target or combination vaccines is an important transmission-blocking strategy. However, this involves the identification of vaccine targets, the determination of vaccine production methods, the correct folding of the corresponding proteins, and the search for relevant vaccine adjuvants. This is a long-term process that requires the long-term delivery of a large workforce and considerable material resources. It is worth noting that there are many endosymbionts naturally present in mosquitoes, and there are also significant differences in malaria parasite infections between different mosquito populations and even regions (Caragata et al., 2020). Therefore, we may be able to directly use these endosymbionts to develop malaria-blocking strategies, which would be extremely beneficial for some current malaria-prone areas. Although there are still some problems to be solved, we believe that this biological blocking strategy would be an important auxiliary strategy to help stop the spread of malaria.

### 6.1 *Wolbachia*

#### 6.1.1 Transmission-Blocking Strategies

**Figure 2:**
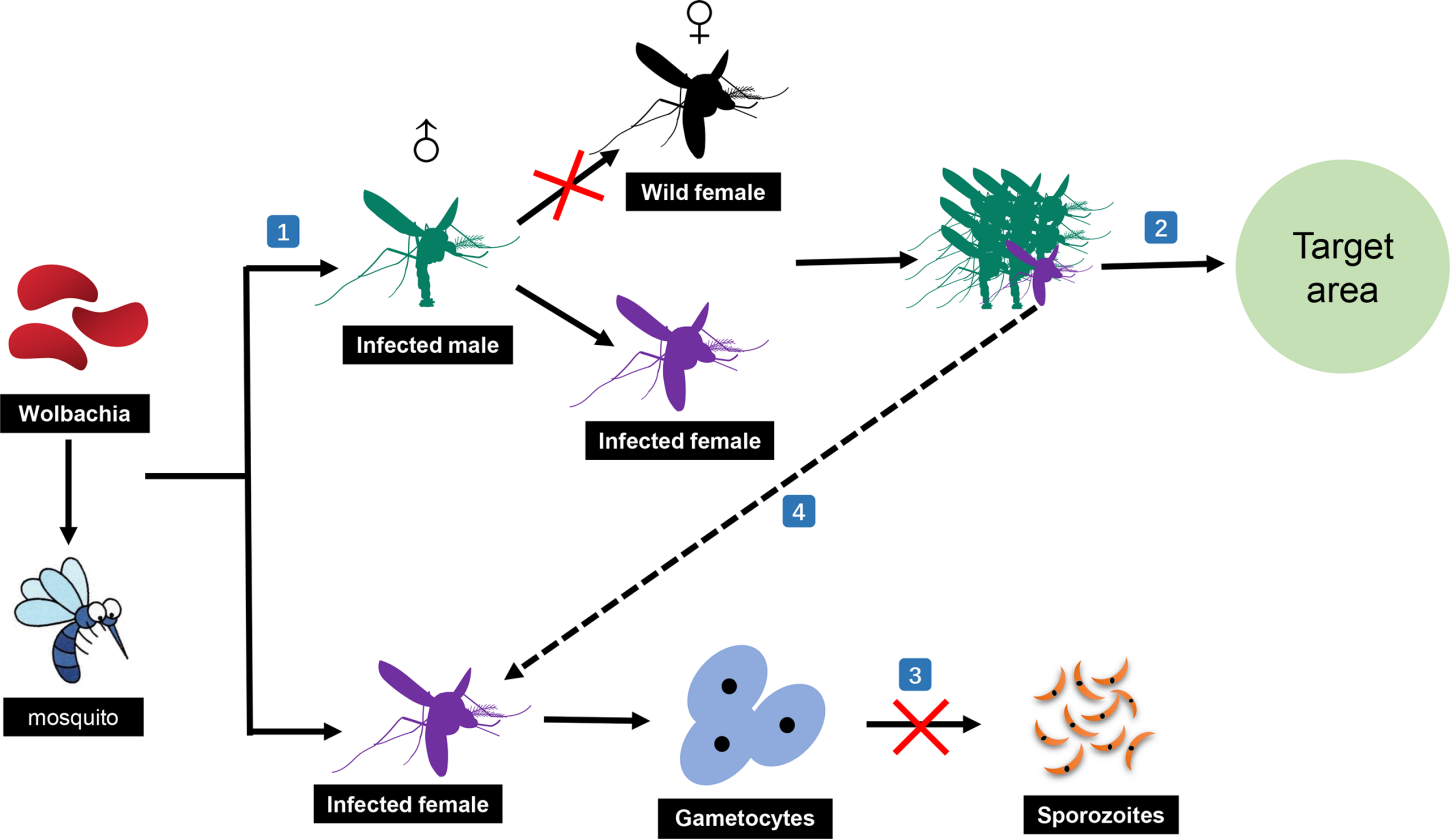
Malaria transmission-blocking strategies based on *Wolbachia*. (1) Male mosquitoes infected with *Wolbachia* can only mate with female mosquitoes infected with the same *Wolbachia* to complete normal reproduction. (2) Numerous infected male mosquitoes can be released in the target area to kill the mosquitoes in the area and thus stop the spread of malaria. (3) *Wolbachia* can also prevent the normal sporozoites development of *Plasmodium* in mosquitoes. (4) Infected female mosquitoes accidentally released with the numerous infected male mosquitoes can still block the spread of malaria through the pathogen suppression ability of *Wolbachia* itself.

*Wolbachia*, as a potential bio-replacement strategy, has received significant attention in the control of malaria in recent years (Dahmana and Mediannikov, 2020). This is mainly owing to the benefits of *Wolbachia* infections, which induce mosquitoes to produce two desirable properties for disease vector control, cytoplasmic incompatibility (CI) and pathogen inhibition (Walker et al., 2021). Meanwhile, there is increasing evidence that *Wolbachia* infections in mosquitoes are common (Baldini et al., 2014; Niang et al., 2018; Jeffries et al., 2021); 25 species of African *Anopheles* mosquitoes have been found to carry 16 varieties of *Wolbachia* infections (Ayala et al., 2019). The desirable induction properties and diverse flora have prompted researchers to further increase their interest in using *Wolbachia* for developing biological control strategies against malaria transmission (**Figure 2**).

Overall, there are two main biological control strategies based on *Wolbachia*: (1) using CI to kill mosquitoes and (2) relying on the transmission-blocking ability of *Wolbachia* in the mosquito stage of *Plasmodium* development. Through CI, *Wolbachia*-infected females produce viable *Wolbachia*-infected offspring when they mate with uninfected males or males infected with the same *Wolbachia* strain. However, *Wolbachia*-infected males only produce viable offspring when they mate with females infected with the same *Wolbachia* strain. Meanwhile, the molecular mechanism of CI has been recently elucidated: CI causes embryonic death when a male expressing the prophage WO genes, *cifA* and *cifB*, mates with an uninfected female or a female infected with an incompatible *Wolbachia* strain. In contrast, embryonic development could be rescued in mosquito females harboring a compatible *cifA*-expressing strain (LePage et al., 2017; Shropshire et al., 2021). In the last five years, based on these properties, various research groups have explored whether they can release a large number of *Wolbachia*-infected male mosquitoes to prevent egg hatching, resulting in adult mosquito numbers being significantly reduced in the target area (Mains et al., 2016; Kittayapong et al., 2018; Kittayapong et al., 2019; Mains et al., 2019; Zheng et al., 2019). Surprisingly, all experiments acquired positive results, providing good research models for subsequent studies.

However, before promoting the CI-based mosquito population control strategy, several key issues need to be resolved. The first is the costs associated with rearing and releasing large number of mosquitoes. The second is that the target population may recover over time after release is halted (Caragata et al., 2020). These pitfalls may need to be dealt with using cost–benefit calculations and sophisticated monitoring. More critically, there is no guarantee that there would be no female mosquitoes released along with the males; thus, there are concerns that accidentally released female mosquitoes may quickly replace the mosquito population in the target area, rendering future suppression releases less effective. Recent studies have attempted to overcome this issue by using aspects of the sterile insect technique during mosquito rearing, with pupae irradiation before release, and positive results have been obtained (Kittayapong et al., 2018; Moretti et al., 2018a; Moretti et al., 2018b; Zheng et al., 2019). However, more efficient male and female separation technology is the focus of further research. In addition, to obtain the most benefit, the timing of the release of the mosquitoes may also need to be considered, although there have been fewer studies along these lines (Huang et al., 2020).

*Wolbachia* itself also has certain transmission-blocking effects on the malaria parasite. It has been demonstrated to have a strong harmful effect on sporozoites and could significantly reduce the number of sporozoites in the mosquito stage (Gomes et al., 2017). The current understanding is that this effect may involve the influence of *Wolbachia* on the mosquito immune system, nutritional competition, mosquito lifespan, and its interaction with the mosquito microbiome (Kambris et al., 2009; McMeniman et al., 2009; Moreira et al., 2009; Audsley et al., 2018). Studies have shown that anti-*Plasmodium* immune genes, including TEP1, LRIM1, Toll pathway gene Rel1, and the effector Defensin 1, were induced by *Wolbachia* in *Anopheles stephensi* mosquitoes (Joshi et al., 2017). However, another study on *Aedes fluviatilis* revealed that the immune activation effects did not seem to include changes in Toll or IMD immune gene transcription (Caragata et al., 2017). Thus, accidentally released female mosquitoes would not be useless. This is another strategy that uses *Wolbachia* to block malaria in mosquitoes. The advantage of using this strategy is that it requires a relatively small number of mosquitoes to replace the original population in the target area owing to the breeding advantage of the new mosquitoes (O’Neill et al., 2018; Zélé et al., 2018; Ryan et al., 2019). However, it is still necessary to consider the bite problem of female mosquitoes and the possible consequences. Although there have been no reports of using this strategy to block the spread of malaria, good progress has been made in blocking dengue fever (Nazni et al., 2019; Ryan et al., 2019). The identification of the actual effect of this strategy in blocking the transmission of malaria requires follow-up studies.

#### 6.1.2 Restrictions on Actual Application

When using *Wolbachia*-infected mosquitoes to develop malaria transmission-blocking strategies, the most critical issue is that the symbiont should establish a stable relationship with the insect carrier, that is, long-term effective vertical or horizontal transmission (Bian et al., 2013; Wilke and Marrelli, 2015; Walker et al., 2021). Although some stable *Wolbachia*-infected mosquitoes have been established through embryonic injection followed by transgenerational spread (Dobson et al., 1999; Sinkins, 2004; Bourtzis et al., 2014), it is still necessary to understand how *Wolbachia* establishes a symbiotic relationship with mosquitoes to develop a more efficient and stable infection plan.

The use of innate immunity to establish a stable symbiotic relationship with mosquitoes is an important mechanism of *Wolbachia* infection. Generally, when *Wolbachia*-free insects are artificially infected, an antimicrobial immune response is triggered to eliminate *Wolbachia*. However, *Wolbachia* could prevent this elimination by evading the AMP immune response or suppressing autophagy-related immune defenses (Zug and Hammerstein, 2015). Meanwhile, another study showed that inhibiting the IMD pathway alone or simultaneously inhibiting the Toll and IMD pathways by RNA interference could significantly reduce the *Wolbachia* load in mosquitoes, although the specific mechanism still needs to be clarified (Pan et al., 2018).

In addition to regulating the mosquito’s immune system, the complex interaction between *Wolbachia* and the mosquito’s natural microbiota is another aspect of the mechanisms underlying the stability of this infection. Certain species of mosquitoes are not naturally infected with *Wolbachia*. When a *Wolbachia* infection is introduced artificially, it is unsurprising that significant vertical transmission barriers are observed, making it difficult for this infection to spread to the next generation. However, when antibiotics continue to be used to treat these mosquitoes, a significant increase in the titer of *Wolbachia* infection was observed, thus achieving perfect maternal vertical transmission (Hughes et al., 2014). Subsequent studies have also shown that some bacterial groups were negatively related to *Wolbachia* infections in mosquitoes, and Asian symbionts have been experimentally confirmed to limit the vertical transmission of *Wolbachia* in mosquito populations (especially infection in the reproductive tissues) (Hughes et al., 2014; Rossi et al., 2015; Straub et al., 2020). Moreover, it is worth noting that the invasion of malaria parasites into mosquitoes would also affect the bacterial composition in the mosquito’s midgut (Sharma et al., 2020), suggesting that related research on *Wolbachia* may deepen our understanding of malaria and thus promote the development of transmission-blocking strategies.

Meanwhile, as the antimalarial ability of *Wolbachia* is controlled by itself but not manipulated by humans; apart from understanding how it infects parasites, we also need to explore whether pathogen escape occurs under infection conditions. To date, although no evidence of pathogen escape was found in wild populations of *Wolbachia*, pathogens seem to be able to survive under infection by certain laboratory strains, suggesting that we need to remain vigilant and take precautions (Pimentel et al., 2021).

The last thing that may require attention is how to determine the presence of *Wolbachia* infections in mosquitoes. At present, most studies use nested PCR tests to detect infections; however, they are limited to amplifying only a few genes (especially 16S rRNA). This is problematic given the possibility of amplifying prokaryotic 16S rRNA genes from non-living cells (Carini et al., 2017; Chrostek and Gerth, 2019). Therefore, more robust evidence is required to determine whether *Wolbachia* strains are established as endosymbionts in *Anopheles* species (Chrostek and Gerth, 2019). Several recent studies have confirmed the existence of natural infection by observing *Wolbachia* infection in the ovaries of mosquitoes over several generations (Ross and Hoffmann, 2021; Walker et al., 2021), but this method is less efficient and is not suitable for vigorous promotion. Thus, a more efficient and convenient method is still urgently needed. Furthermore, studies have shown that different types of *Wolbachia* may have different effects on malaria parasite infection in mosquitoes. It has been reported that infection with certain strains of *Wolbachia* can increase malaria parasite infection in mosquitoes (Hughes et al., 2012; Murdock et al., 2015). Overall, there are still many problems that need to be overcome when using *Wolbachia* infections to block the spread of malaria.

### 6.2 Intestinal Flora

During the development process of malaria parasites in mosquitoes, in addition to genetic variation, the intestinal microbiota also makes an integral contribution to the susceptibility of mosquitoes to infection. After the malaria parasite enters the mosquito body, it makes its way to and settles in the midgut; thus, there must be numerous complex interactions between the intestinal microbial colony and the malaria parasite. In fact, there is already evidence showing a strong connection. Previous opinions were that malaria fever prevented bacterial infections by halting the synthesis of lipopolysaccharides, thereby interfering with the growth of bacteria, but the actual situation suggests that the coexistence of malaria and bacterial infections, such as gram-positive *Staphylococcus aureus* and gram-negative *Escherichia coli* infections, is common in the tropics where malaria is endemic. The intestinal microbiota has also been found to comprise a higher quantity of *Bifidobacterium* and *Streptococcus* species in healthy individuals than in *P. falciparum*-infected patients (Sahar Traoré et al., 2015). In addition, some microorganisms have also been shown to significantly affect the ability of mosquitoes to act as vectors by regulating the immune response in the mosquito body (Gabrieli et al., 2021). These results indicate that it would be beneficial to develop malaria transmission-blocking strategies, and even some vaccines, based on the intestinal flora.

However, studies on how the intestinal flora affects malaria parasites in mosquitoes are relatively rare. Most research has focused on the relationship between the clinical symptoms of malaria and the intestinal flora (Ashour and Othman, 2020). Despite this, the studies conducted so far have provided a good reference and guide for future in-depth studies. The co-feeding of *E. coli*, *S. aureus*, and *P. falciparum* gametocytes has been shown to significantly reduce the malaria parasites infection level in mosquitoes (Gabrieli et al., 2021) and secretions from some flora may play key roles in the specific mechanism of this inhibition. Romidepsin, a protein secreted by the intestinal flora, has been shown to limit *Plasmodium* infection in rodent animals (Saraiva et al., 2018). A strain of *Enterobacter* isolated from wild *Anopheles arabiensis* mosquito populations in Zambia has also been shown to interfere with the development of malaria parasites *via* the production of reactive oxygen species before they invade the midgut epithelial cells (Cirimotich et al., 2011). Such flora may also control *Plasmodium* infection by regulating the expression of some genes in the midgut of mosquitoes. A previous study indicated that the intestinal flora could increase the expression of TEP1 protein in the midgut of mosquitoes in a manner similar to RNAi silencing to promote *Plasmodium* infection, but the specific mechanism remains a mystery (Wang et al., 2013).

## 7 Discussion

Although malaria prevention relies mainly on insecticide-treated bed nets and indoor residual spraying, major breakthroughs have been made in the prevention and control of malaria in the past decade. However, the number of malaria deaths worldwide has remained stable at around 400,000/year in recent years, which is a crucial fact that drives the development of global malaria prevention and control (Caragata et al., 2020). ACT and a variety of other preventive drugs, including primaquine, were seen as a new dawn in reducing global malaria deaths. However, the increasing number of drug-resistant strains has greatly impacted the effectiveness of these drugs and has become a great obstacle for local malaria prevention and control measures (Wang et al., 2021). Although many studies aim to respond to the current urgent situation by developing new antimalarial drugs or modifying existing drugs, because of the long cycle of drug development and other problems, there is currently no new drug that can resolve this situation. In addition, for many countries or regions, it is not simply a case of reducing the incidence of malaria, as the total eradication of malaria is their end goal. Under these conditions, in addition to the use of antimalarial drugs, it would be beneficial to find other more promising ways to block the spread of malaria in the natural environment, thus eliminating the possibility of malaria infection.

Unlike clinical malaria, the reproductive period of the malaria parasite in mosquitoes is key to its widespread transmission. An obvious benefit is that, if the propagation of malaria parasites in mosquitoes is blocked, not only can the infection of the population be reduced, but more importantly, it can also prevent the spread of existing drug-resistant strains, thereby prolonging the effectiveness and use of antimalarial drugs. After the entry of the malaria parasite gametocytes into the mosquito, they undergo the following sequential steps: leaving the host red blood cell, the conversion of the male and female gametocytes, fertilization, zygote invasion of the mosquito’s midgut epithelium, and the subsequent oocyst development stages. Numerous studies have shown that several proteins from the different stages can be utilized as candidate targets for TBVs; however, there are still many difficulties to be overcome. For gametocyte surface proteins, only a small number of candidate proteins have been identified. What needs to be considered is how to produce these recombinant antibodies correctly and efficiently. Also in the future, the number of candidate target proteins can be increased through detection in areas of low-prevalence (Paul et al., 2016). In current research focusing on fertilization-related processes, whether interfering with the conversion of gametocytes to male and female gametocytes or with the combination of male and female gametocytes, excellent results have been achieved. However, the lack of innate immunogenicity of these proteins is an unavoidable problem, and a highly effective adjuvant without adverse side effects is essential for their practical use. Some scholars have suggested that, under the pressure of TBV, the ability of *Plasmodium* to increase male gametes to ensure transmission may be a test for the effectiveness of vaccines targeting male gametes (Teboh-Ewungkem et al., 2021). Further research is needed to clarify the specific mechanism for this. The targets in the post midgut developmental stage are different from those in the first two stages. The candidate proteins used can either be from the malaria parasite or from the mosquito’s midgut. Therefore, a study attempted to genetically modify mosquitoes to block the development of malaria parasite oocysts in the midgut. Although there was a significant reduction in the development of the oocysts of the malaria parasite in the mosquito, it also caused a significant physiological burden on the mosquito itself (Dong et al., 2018). This suggests that in the direct genetic modification of mosquitoes, we need to be mindful of the impact on the physiological characteristics of the mosquito itself and their impact on the ecosystem. Moreover, the relevant antibody will only provide a transmission-blocking effect in the mosquito when the mosquito sucks the blood of a recipient vaccinated with the TBV. Thus, some scholars have explored the possibility of a vaccine that either has a transmission-blocking ability or a benefit for clinical malaria during the period that it exists in the blood of the recipients. A whole-killed blood-stage vaccine may be worth exploring, as it not only provides protection against the blood-stage challenge, but also significantly inhibits the development of malaria parasites in mosquitoes with the help of parasite-specific IgG and the inflammatory cytokine MCP-1 (Zhu et al., 2016). Furthermore, there are two issues that need to be addressed. A multi-target vaccine for the reproductive phase did not achieve synergistic effects in previous experiments (Zheng et al., 2016). Researchers believe that it may interfere with the specificity of the antigen response, but the specific mechanism remains unclear. In addition, during the development of malaria parasite vaccines, the correct folding and modification of the protein should be studied, as this is crucial for determining whether it can correctly cause a vaccine response (Patra et al., 2015).

The transmission and infection of *Plasmodium* is not only a two-way effect between the host and the parasite but also involves many different microorganisms which play an important role. There is increasing evidence that these microorganisms could help significantly block the spread of *Plasmodium* parasites. *Wolbachia*, an endosymbiont that is widespread as a parasite in mosquitoes, has been shown to play an important role in blocking malaria transmission (Ross and Hoffmann, 2021). Owing to its reproductive characteristics during its parasite phase, the release of male mosquitoes infected with *Wolbachia* can directly kill the mosquito population in the target area. However, it is difficult to ensure that female mosquitoes are not also released. This presents a need for more stringent requirements in male and female separation technology (Caragata et al., 2020). *Wolbachia* itself can also inhibit the growth and development of *Plasmodium* in mosquitoes, although the specific mechanism is still unclear. In addition, the intestinal flora seems to be a potential target for blocking the transmission of malaria, but there are currently only a few relevant studies. The advantage of applying these biological control strategies is that they are not as complicated as vaccine development, and the technical requirements are lower. This suggests that biological control strategies may be practically applied as malaria control measures in the short term to address the current situation. Even so, we still need to consider the possible impact of these newly introduced organisms on the local ecology, devise possible response plans, and provide possible new alternative control strategies (Bellini et al., 2020).

What needs to be carefully considered if TBVs are used to block the transmission of malaria, is the frequency of vaccination, which should be determined to ensure that the antibodies can be maintained at an effective level. This requires numerous studies to determine the effective concentrations of the relevant antibodies. Meanwhile, there is currently no relevant research exploring whether TBVs have side effects in the human body. These are problems that should be resolved in the future. In addition, if *Wolbachia* is used to kill mosquitoes to stop the spread of malaria, the effects on the local ecosystem must be clarified. In general, although there is still a long way to go before their practical use, the development of TBVs and biological control strategies based on endosymbionts could significantly inhibit the widespread distribution of malaria. These directions show promise for alleviating the current malaria situation and assisting the end goal of malaria eradication.

## Author Contributions

YW and SY conceived and designed the study. SY wrote the draft of the manuscript. JW, XL, HZ, LW, XY, and YW improved all the versions. All authors contributed to the article and approved the submitted version.

## Funding

This study was supported by the National Natural Science Foundation of China (81971971 and 81702035), the Scientific Research Project of Army Medical University (2021XJS05), the Graduate Scientific Research Innovation Project of Chongqing China (CYB20191) and the Natural Science Foundation of Chongqing China (cstc2018jcyjAX0182).

## Conflict of Interest

The authors declare that the research was conducted in the absence of any commercial or financial relationships that could be construed as a potential conflict of interest.

## Publisher’s Note

All claims expressed in this article are solely those of the authors and do not necessarily represent those of their affiliated organizations, or those of the publisher, the editors and the reviewers. Any product that may be evaluated in this article, or claim that may be made by its manufacturer, is not guaranteed or endorsed by the publisher.
